# Exploring clinicians’ experiences and perceptions of end-user roles in knowledge development: a qualitative study

**DOI:** 10.1186/s12913-021-06955-7

**Published:** 2021-09-06

**Authors:** Leslie Verville, Carol Cancelliere, Gaelan Connell, Joyce Lee, Sarah Munce, Silvano Mior, Robin Kay, Pierre Côté

**Affiliations:** 1 Ontario Tech University, Oshawa, ON Canada; 2 Canadian Memorial Chiropractic College, ON Toronto, Canada; 3 KITE-Toronto Rehabilitation Institute-University Health Network, Toronto, ON Canada

**Keywords:** Integrated knowledge translation, Clinical care pathway, Qualitative inquiry

## Abstract

**Background:**

End-user involvement in developing evidence-based tools for clinical practice may result in increased uptake and improved patient outcomes. Understanding end-user experiences and perceptions about the co-production of knowledge is useful to further the science of integrated knowledge translation (iKT) – a strategy for accelerating the uptake and impact of research. Our study had two main objectives: (1) explore end-user (clinician) experiences of co-producing an evidence-based practice tool; and (2) describe end-user perceptions in knowledge development.

**Methods:**

We used a qualitative study design. We conducted semi-structured interviews with clinicians and used a transcendental phenomenological approach to analyze themes/phenomena. In addition, we explored the interrelated themes between the thematic maps of each objective.

**Results:**

Four themes emerged from clinicians’ experiences in co-producing the practice tool: ease/convenience of participating, need for support and encouragement, understanding the value of participating, and individual skillsets yield meaningful contributions. Stakeholder roles in knowledge tool development and improving dissemination of evidence and knowledge tools were themes that related to clinician perceptions in knowledge development. The review of interrelated thematic maps depicts an intertwined relationship between stakeholders and dissemination.

**Conclusions:**

End-users provide invaluable insight and perspective into the development of evidence-based clinical tools. Exploring the experiences and perceptions of end-users may support future research endeavours involving iKT, such as the co-production of clinical resources, potentially improving uptake and patient health outcomes.

**Supplementary Information:**

The online version contains supplementary material available at 10.1186/s12913-021-06955-7﻿.

## Background

Involving end-users in developing evidence-based tools for clinical practice may result in better uptake and improved patient health outcomes [1–4]. Understanding end-user experiences in the co-production of knowledge is useful to further the science of integrated knowledge translation (iKT) – a strategy for accelerating the uptake and impact of research.

iKT promotes the collaboration of researchers and end-users contributing to mutually beneficial outcomes [5, 6]. iKT emphasizes end-users participating throughout the knowledge development process, thereby maximizing the *accessibility* (ability to understand and apply the research), *relevance* (applicability of the research questions to current concerns), and *endurance* (sustainability of changes associated with the research findings) of research outcomes [5, 7–11]. End-users are often involved in research in a consultation capacity through interviews, surveys, or review committees [12]; however, there is limited evidence to support the efficacy of these approaches [2, 13] It is unclear whether these approaches allow researchers to capitalize on the benefits of end-user involvement. Therefore, work is needed to guide how to incorporate the contributions of end-users into the research comprehensively [12, 14–17]. Specifically, there is a need to explore the nature of the collaboration between researchers and end-users to help support end-user engagement, particularly with respect to rehabilitative healthcare [18–23]. Exploring end-user experiences and perspectives in participating in knowledge development may improve the quality of the methodology and optimize the research outcomes such as utilization and relevance [1, 2, 4, 12, 23].

Our study is situated within a larger project, whereby end-users co-developed an evidence-based practice tool (i.e., online clinical care pathway to facilitate the clinical management of soft-tissue shoulder pain in adults) with our team of researchers [24] The end-users’ key contribution was providing an understanding of end-user needs and preferences (e.g., current practice patterns, knowledge gaps, information trends, preferred tool formatting) and barriers of practice guideline implementation (e.g., patient, professional, organizational, system, economic, political, or social/cultural factors). In the current study, we aimed to (1) explore end-user (clinician) experiences of co-producing an evidence-based practice tool; and (2) describe end-user perceptions of participating in knowledge tool development.

## Methods

### Study Design

**Fig. 1:**
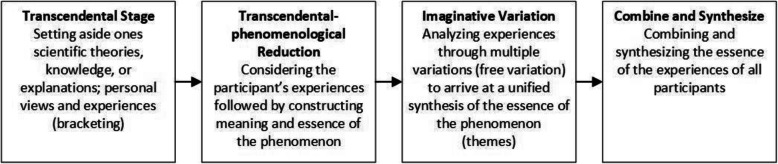
Transcendental phenomenological approach. Footnote: Bracketing: identification and temporary suspension of researcher assumptions [28]. Free variation: imagining the phenomenon in a variety of ways/contexts to develop an understanding of the essence of the phenomenon [25].

We conducted a qualitative study using a transcendental phenomenological approach using a constructivist lens (Fig. 1) [25–27]. This approach aims to understand the essence of a phenomenon whereby the researcher aims to achieve a bias-free state of mind and relies on the objective interpretation of participants’ lived experiences [25].

We obtained ethics approval through the Research Ethics Board of Ontario Tech University (REB #15,436). All methods were performed in accordance with the relevant guidelines and regulations. We used the Consolidated Criteria for Reporting Qualitative Research (COREQ) to guide this study’s conduct and reporting [29].

### Participants and Recruitment

Through our professional networks, we invited clinicians to develop the care pathway with our team of researchers and then to subsequently participate in a semi-structured interview to meet our current research objectives. We invited clinicians from disciplines that commonly manage patients with shoulder pain (i.e., chiropractors, medical physicians, physiotherapists). We used purposeful maximum variation sampling to achieve diversity in years of practice, educational background, practice characteristics (multidisciplinary vs. solo), and geographical representation (Canada, United States) to promote the transferability or our results. Clinicians were contacted via email and sent an invitation letter. Recruitment extended from May 2020 to October 2020 until data saturation was reached, defined as the point at which no new useful information relative to our study objectives was obtained for two consecutive interviews [30].

A ‘Think Aloud’ protocol was used to capture clinicians’ initial perceptions about the care pathway. Clinicians were presented with a clinical vignette and asked to independently consider it while applying the online care pathway. They were asked to speak out loud (to their computer) about their actions, followed by answering a series of questions: (1) what did you like about the care pathway? (2) what didn’t you like about the care pathway? (3) was there enough information in the care pathway to inform your clinical decision-making? (4) how would you improve the care pathway? and (5) how would you use the care pathway in your practice? The ‘Think Aloud’ protocol was recorded using the screen recording program, Loom [31]. Feedback provided by clinicians was used to modify the care pathway accordingly.

### Semi-structured interviews

We conducted online individual semi-structured interviews using Zoom after developing the care pathway. Interviews explored participants’ experiences with participation and perceptions of their roles in knowledge tool development. We used a semi-structured interview methodology because of its versatility and flexibility enabling reciprocity between the interviewer and interviewee, lending to a more natural flow of conversation while ensuring key points are explored [32, 33].

A trained interviewer led all interviews (GC) [credentials: BHK, DC; occupation: knowledge broker; sex: male]. Training included concepts in limiting personal biases throughout the interview such as active listening without demonstrating emotional reactions to interviewee responses [34]. An additional female researcher was present during each interview to support the primary interviewer when needed and write field notes (LV, JL). Participants were not aware of the interview questions prior to the interview (Additional File 1). Interviews lasted approximately 45 min and were audio-recorded. Recordings were saved as audio files and transcribed verbatim by an external transcriptionist. No repeat interviews were conducted. Identifying information was removed from the transcripts prior to analysis.

## Analysis

We used a transcendental phenomenological approach to conduct our analysis. In addition, we adopted the six-phase conceptual framework by Braun and Clark (2006) to support the Imaginative Variation stage of our approach: (1) familiarizing yourself with the data; (2) generating initial codes; (3) searching for themes; (4) reviewing themes; (5) defining and naming themes; and (6) producing the report [35]. While this framework is presented in phases, this process was not conducted linearly. We revisited phases throughout the analysis process to facilitate a thorough interpretation of the data [35].

An initial open coding scheme with definitions was developed *a priori* based on our research objectives and the interview guide to provide reviewers with an initial list of codes to facilitate coding of transcripts (Table 1) [36]. Following each interview, clinicians were offered an opportunity to review their transcripts to ensure trustworthiness. Clinicians validated the transcripts by correcting and clarifying them as necessary [37].

**Table. 1:** Organizational coding tree

Clinician	Pertaining to the clinician being interviewed
Experiences with participation	Pertaining to the clinician’s experience with the present study
Care pathway	Pertaining to the knowledge tool developed
Knowledge translation	Pertaining to knowledge translation activities

Data were analyzed throughout the data collection period. Following each interview, three reviewers independently coded the transcript. Independent coding of transcripts was used to ensure reliability of interpretation. Reviewers independently worked systematically through the data, extracting new codes to coincide with emergent themes. The reference coding scheme and definitions were revised as axial coding themes emerged [36]. Consensus was achieved through discussion. Subsequent transcripts were coded using the continually revised coding scheme. Once the coding scheme was revised, previous transcripts were re-coded according to the updated scheme. This process was continued until all transcripts were coded. Saturation was assessed following the consensus of each transcript and was determined when no new themes emerged for two consecutive interviews.

We used NVIVO v.11 to organize the codes and quotes from the transcripts. We created mind maps with headings representing our two research questions: participant experiences (1) and clinician perceptions in knowledge development research (2). Transcript codes and affiliated quotes were grouped into themes. Broad themes were linked to sub-themes to describe overall phenomena related to our questions. Themes were sequenced and linked to one another, creating a ‘web’ of thematic connections. We implemented member checking to improve the trustworthiness of our analysis [38]. All clinicians were asked to review and provide feedback about the mind maps, themes, and definitions to ensure our findings’ correctness and credibility. Summary statements were developed to describe the overall phenomena for each research question supported by themes and clinician quotes. We selected representative quotes that most articulately described each theme.

We also conducted an analysis of interrelated themes; thematic connections that further integrate themes. We juxtaposed the mind maps from both research questions and identified thematic connections between them by comparing and relating themes to one another, creating an interpretation of the overall meaning [36, 39]. Summary statements were developed to describe the overall connected phenomena.

## Results

We invited 18 clinicians to participate (nine chiropractors, three physiotherapists, and six medical physicians). Twelve clinicians consented, and nine participated in the knowledge tool co-production and subsequent interviews. We were unable to obtain reasons for loss to follow-up. We achieved saturation of themes within the participant sample.

The majority of participating clinicians were chiropractors (89 %); one participant was a medical physician. No physiotherapists participated. Most clinicians were male (89 %), and the mean age was 46 years [SD 15.2]. In addition, the majority of clinicians indicated that they practice in Canada (8/9), with a mean number of years in practice of 16 (SD 13). Most attained their professional degrees in Canada (78 %), and 67 % reported having an advanced degree (e.g., master’s degree, Ph.D.).

All participants were invited to review their transcripts and resultant findings. One participant edited their transcript, and four provided edits to the mind maps, themes, and definitions.

**Fig. 2:**
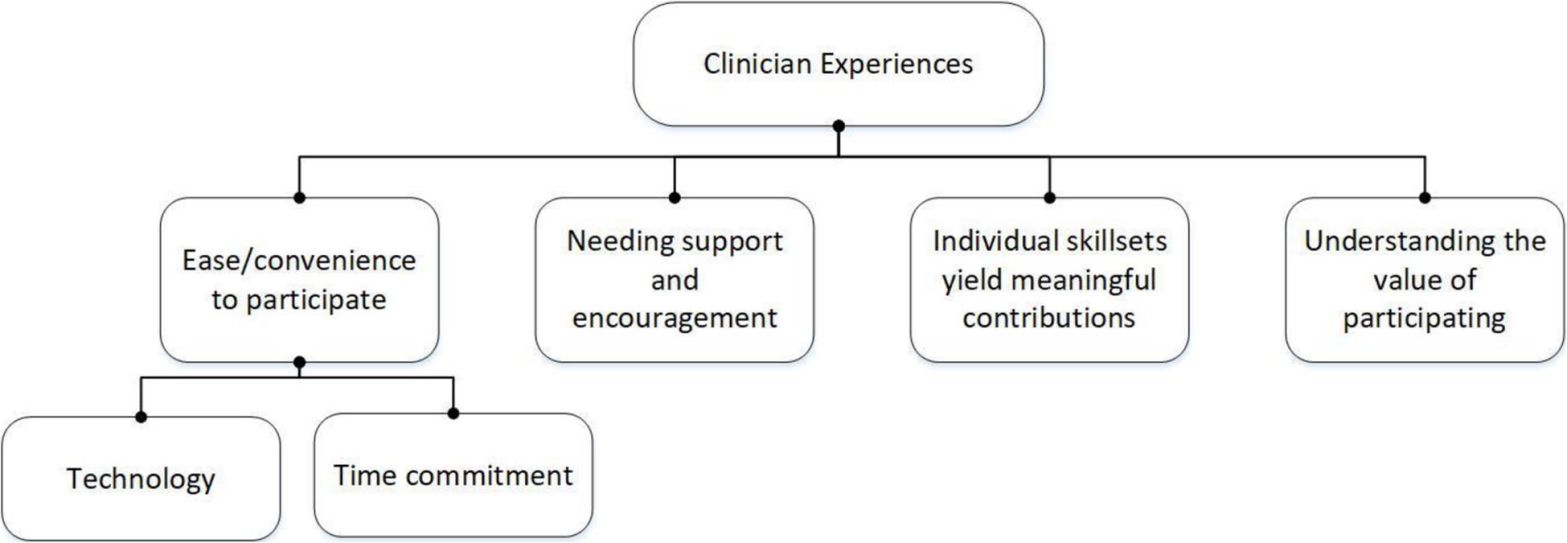
Clinician experiences co-producing an evidence-based practice tool for the management of shoulder pain

Four themes emerged from clinicians’ experiences in co-producing the practice tool: ease/convenience of participating, need for support or encouragement, understanding the value of participating, and individual skillsets yield meaningful contributions (Fig. 2).

### Theme 1: Ease/convenience of participating

This theme describes the conflicting perceptions about the ease/convenience (feasibility) expressed by participants during their participation in the Think Aloud activity using the screen capture recording program (Loom). Clinicians reported that the time commitment required to participate was suitable and not too onerous.

*“What was asked of me wasn’t too onerous, just took some energy” –Clinician 7*.

There were conflicting ideas about using technology to participate. Some clinicians found the screen capture program (Loom) straightforward and easy to use, while others found it challenging and had reservations about downloading a program to record themselves (i.e., they were not sure if the program would record them unknowingly at a later date).

*“The program [Loom] was straightforward to use” –Clinician 9*.

*“It was a bit more complicated to get set up than I think is ideal…do I really want this thing on my computer, is this going to be activated at some other time and record something else I am doing? I mean, I think there would be a natural thought about I don’t really want this program necessarily on my computer and that absolutely occurred to me.” –Clinician 3*.

### Theme 2: Need for support and encouragement

This theme captures the clinicians’ perspectives that their contribution could have been strengthened if they were provided more timely and effective feedback throughout the process. Clinicians sought assurance that they were participating ‘correctly’. Clinicians felt that the instructions for participation in the independent Think Aloud activity was lacking and that they did not feel confident that they were contributing to the project to their full capacity because of this limitation.

*“So, as I was doing it most of what I was doing was thinking am I doing this right? Is this what they want? And then I’m not so sure if I got to that end product that you guys were looking for” –Clinician 2*.

### Theme 3: Understanding the value of participating

This theme captures clinicians expressing interest in having a clearer understanding of how their involvement contributed value to, and helped inform the development of the care pathway. Specifically, they wanted to know the value of their contributions and in what capacity it would influence the results of the project.

*“I wish I knew more about how my comments and review of the care pathway would be used and if I would be informed of this or not.” –Clinician 5*.

### Theme 4: Individual skillsets yield meaningful contributions

The individual clinician skillsets, such as clinical background and experience, were perceived to enhance their contribution to the development of the care pathway. Clinicians felt these areas helped them contribute meaningfully to the overall outcome of the project.

*“My experience as a clinician helped to inform what the pathway might look like from beginning to end” –Clinician 3*.

Clinicians who held more than one role (e.g., a clinician who is also an educator) indicated that their contributions could be additionally meaningful by drawing upon these various perspectives.

*“My educator background helped me to identify the sticking points that I would anticipate questions on if I were presenting this in a lecture format. I was able to then address these points during the Think Aloud activity.” –Clinician 2*.

**Fig. 3:**
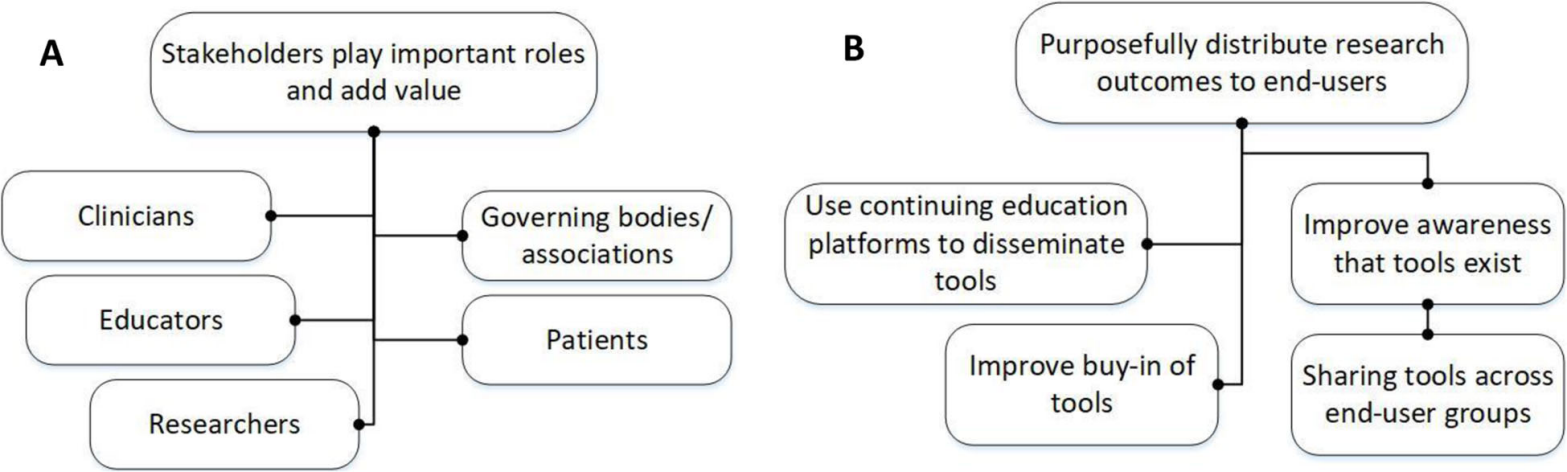
**A**-**B**: Clinician-perceived roles and responsibilities of end-users in knowledge development research projects

Regarding clinician perceptions in knowledge development, two themes emerged: the roles and responsibilities of prominent stakeholder groups and barriers and facilitators of dissemination practices (Fig. 3).

### Theme 5: Stakeholder roles in knowledge tool development

This theme captures the important roles stakeholders play, and their added value in the development of knowledge tools. These stakeholders include clinicians, researchers, and non-clinicians (educators, patients, and governing bodies/associations). Clinicians also discussed the importance of assembling a diverse group of stakeholders representing all of the end-users of research outcomes and knowledge tools.

*“The stakeholder group needs to be representative because what we do know is that when people feel that some of their colleagues are part of this, people they know and respect and are champions and leaders, they are more likely to think that this is for them. So, the likelier amount of uptake is great.” –Clinician 5*.

A clinician’s role as a stakeholder was widely discussed. In their role as stakeholders, it was recommended that clinician groups include diverse backgrounds, ensuring a mix of experience (e.g., years in practice), sex, and clinical expertise. Viewpoints varied regarding the level of participation clinicians should have in knowledge development and implementation. Some participants suggested clinicians should be included in the research team and participate in all phases of the research process. This would include setting the objectives, methods, data collection, and interpretation and dissemination of results (iKT); thereby maintaining relevance for the end-user that otherwise could not be ascertained by researchers alone. Others indicated that clinicians may be better suited to inform the final stages of research (end of grant KT or user testing) due to their lack of research expertise.

*“Clinicians are the ones that are going to ultimately use the information and I found that having them around the table has always been very helpful and that they provide some insights that, once removed from clinical practice, we might not realize.” –Clinician 5*.

*“Clinicians should just be involved in the road testing or beta testing. That is sort of it, like the idea of guidelines is interesting in the sense that it is clinical, it would be more like a clinician would like to obtain it as a tool as opposed to necessarily build the tool. It is giving it the validation of use at the end of it.” -Clinician 1*.

The value that clinicians bring through participating in research and knowledge tool development was perceived as significant. They indicated that their involvement would improve the credibility of the work and ultimately buy-in from other clinicians and end-users. Reassuring clinicians that their participation brought value and integrity to the final product was also important (i.e., ensuring clinicians understand explicitly how their input will be used to influence the research).

*“It is important to make sure the clinicians feel that their opinions are worthwhile because it is their guidelines not the scientist’s guidelines, it is the clinician’s guidelines. The scientist is incapable of doing it. The clinician has to do it and the clinician cannot do it without the patients because a clinician guideline is of no value if the patients don’t buy it.” –Clinician 7*.

Clinicians discussed the expertise of researchers, and how working together fills their knowledge gap. However, others indicated that researchers should play more of a facilitating role in research and manage stakeholders to achieve research outcomes.

*“Researchers’ role should solely be informing stakeholders what the science says. Everything else after that in based on consensus between stakeholders” –Clinician 7*.

Clinicians identified educators, patients, and governing bodies/associations as important non-clinician stakeholders. Clinicians agreed that educators would provide awareness about how information would be academically applied and provide valuable input to the project’s dissemination strategies.

*“I think have some faculty involved helps because there is a level of awareness about how it is being consumed or making sure we have it integrated in the way that you are intending it to be”* –Clinician 3.

Clinicians described patients as important informants to knowledge development due to their unique role as the receivers of healthcare.

*“We need to find out what is important to them [patients]. So is it functional disability or getting back to normal function, is it getting out of pain, like really understanding what is important to a patient might be helpful because that is going to direct your research” –Clinician 6*.

Finally, clinicians discussed the importance of support from governing bodies and associations especially regarding compliance of implementing research outcomes and knowledge tools.

*“I think that if you were to ensure compliance from healthcare providers and support going forward as it evolves you want the governing bodies, the associations in each discipline to be in full support” –Clinician 4*.

### Theme 6: Improving dissemination of evidence and knowledge tools

This final theme portrayed clinicians’ perspectives about the important barriers and enablers, and related recommended strategies for the dissemination of research evidence and knowledge tools to the end-users. Clinicians supported using continuing education platforms such as seminars to spread awareness of new evidence and tools. In addition, clinicians explained the need for a reliable website to visit for current information and longed for services like electronic health record systems to prompt clinicians to use evidence on a case-by-case basis. While this specific example may not be appropriate in all clinical contexts, stakeholder participation and support is important to guide specific dissemination strategies.

*“So, in terms of accessibility I think one of the main things people get caught up in is, it is not the issue of using it per se but finding it. So, we talked about evidence-based clinicians and are they aware of CCGI, are they aware of the resources out there, so I think awareness is big”* –Clinician 8.

One clinician described that evidence and guidelines seem to be updated continuously and therefore indicated they should only be informed of new guidelines if the evidence has changed or a treatment should be abandoned due to changes in evidence. Participants warned about the potentially negative effects of informing clinicians of new evidence too often as clinicians may grow frustrated, feeling they need to constantly change their practice behaviours.

*“I just think awareness is going to be a big thing going forward because there is so much information out there and it is really hard for clinicians to keep on top of everything.” –Clinician 6*.

Clinicians discussed the importance of having all the evidence available to them on a particular topic and not siloed based on professions (i.e., providing evidence only within the scope of chiropractic practice). Providing such evidence ensures that clinicians, regardless of specialty, are well informed of the breadth of existing evidence to provide better care for their patients.

*“In my experience even though we work in groups and work inter-professionally we are still really, really siloed. …So, to try and get it so that one clinician will talk to another clinician about it and hey this is great, I use this tool, you should start using it too” –Clinician 1*.

Clinicians discussed strategies for improving the buy-in of clinical resources. Having a representative sample of professions in the stakeholder group is important to ensure each stakeholder’s values are appropriately represented through to the final product of the research (e.g., care pathway). In addition, buy-in can be improved by supporting interprofessional collaboration. Ensuring the language is suitable across professions supports this collaboration enhancing the dialogue between professions.

*“Well there is a couple reasons why it is important to make the language generalizable to other professions; one, because you all want to walk the walk and talk the talk the same thing if you are trying to treat somebody and you are doing some inter-professional collaboration that is important” –Clinician 6*.

### Interrelated Themes between objectives 1 and 2

**Fig. 4:**
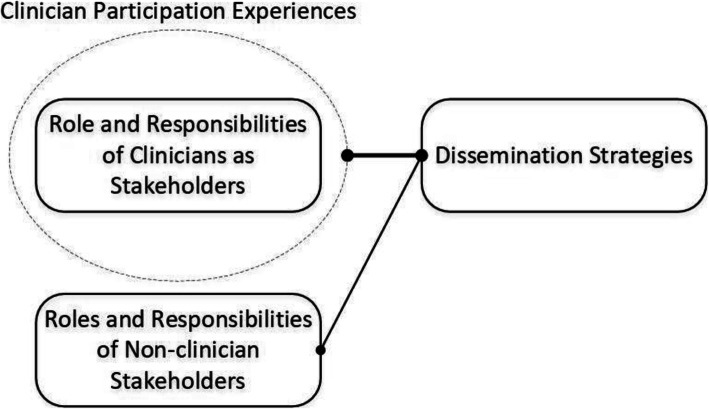
Interrelatedness of stakeholders and dissemination strategies

We noted connections between stakeholders and strategies for dissemination whereby the roles and responsibilities of stakeholders include dissemination (Fig. 4). For example, educators could support awareness of resources within their student population and use continuing education platforms. In addition, governing bodies/associations could support disseminating resources by improving awareness through endorsement and sharing resources across end-user groups. Finally, clinicians can support the dissemination of the research and resources by improving the buy-in of clinicians and patients. Importantly, meaningful clinician participation experience may enhance their role in supporting dissemination.

## Discussion

We described the experiences of clinicians participating in the co-production of a clinical practice knowledge tool as well as clinician-perceived roles and responsibilities of end-users. We found that clinicians generally had a positive experience in participating and indicated that their participation added value and integrity to the project. Clinicians also expressed that their skillsets and, for some, multiple skillsets, played an important role in the meaningfulness and depth of their contribution. Petit-Steeghs et al. (2020) conducted a thematic analysis of interviews with patients and providers participating in the co-creation of a clinical care pathway for sarcoma and gastrointestinal stromal tumours [40]. They identified a power-imbalance between the patients and providers. This imbalance inhibited the meaningful participation due to their varying knowledge gaps of the literature and their understanding of their roles and responsibilities within the research project. Collectively, our findings provide insight into managing end-users and their roles, however, additional work is needed to understand how to maximize this effort comprehensively.

Our results indicate conflicting perceptions toward the specific program used to facilitate the development of the knowledge tool (Loom). Clinicians indicated that they would have preferred additional support to ensure that they were participating ‘correctly’. Further, clinicians also expressed that they did not fully understand how their participation would influence the final knowledge tool. A recent mixed-methods study by Roberge-Dao et al. (2019), aimed to understand the experiences of partnerships between researchers and rehabilitation clinicians participating in iKT projects within a university in Quebec, Canada [23]. This study reviewed 53 rehabilitation-oriented iKT projects aiming to translate knowledge to improve patient health outcomes and quality of care. Clinicians expressed the need for clear, outlined time commitments, funding, and resources to be discussed and agreed upon *a priori* to optimize potential participation. Our findings align with those of Roberge-Dao et al., as clinicians appeared to lack a comprehensive understanding of their role and its impact on the care pathway development.

Further in-depth analyses of end-users’ experiences are required to substantially inform iKT research; to improve the quality of the collaboration by ensuring end-users feel they were able to participate in the research to the fullest of their capacity [5, 41–43]. Our analysis of clinician experiences contributes to addressing this knowledge gap by demonstrating relationships between clinicians’ experiences and their perceived roles and responsibilities. Future research should consider comparing the experiences of multiple stakeholder groups.

With regard to clinician-perceived roles and responsibilities of end-users, clinicians indicated a number of stakeholder groups that would add value to research and knowledge development of this nature; further, a comprehensive end-user group would be optimal. Clinician participation unveiled strategies for dissemination such as using continuing education platforms, improving awareness, sharing resources equally, and improving buy-in. While not explored in depth, tension appeared between clinicians and researchers regarding their roles and value to the research. Future studies should consider exploring this paradigm as a barrier to participation.

### Interrelated Themes

Themes appear closely integrated between both study aims. By juxtaposing the thematic maps, we formed a multidimensional analysis of the experience of clinicians’ participation and the conceptual roles and responsibilities of end-users. Therefore, we can more clearly interpret the breadth of participation achieved and components requiring refinement. Interrelatedness was also present between stakeholder groups and dissemination strategies, whereby specific stakeholders may be integral to the success of certain strategies (e.g., educators improving awareness). Participation experiences appear to optimize clinician roles and responsibilities by identifying barriers and enablers of engagement. Additional connections and interpretations may still exist. For example, the experiences of other stakeholder groups, such as educators or patients, may further our ability to optimize their roles and responsibilities leading to more targeted and fruitful dissemination. Overall, our interrelated analysis appears to depict the intertwined relationship between stakeholders and dissemination.

### Recommendations for improving the experience of end-user participation

1. Ensure instructions and time commitment required of end-users is well understood prior to participation.
2. Encourage end-users to explore how their individual skillsets can meaningfully inform the project objectives.
3. Clearly demonstrate the value end-user participation will have on the overall project outcomes.

### Strengths and Limitations

Our study has strengths. First, we followed a sound qualitative methodology and theoretical framework to guide the conduct of our study. Using this framework, themes were sequenced and linked to create a web of thematic connections; broad themes linked to sub-themes insightfully describing the overall phenomena for each of our research objectives. We implemented two forms of member checking to improve the trustworthiness of our data and externally audited our analysis.

Our study also has limitations. We were unsuccessful in recruiting a balanced sample across professions involved in the clinical management of shoulder pain in adults. This likely impacts the transferability of our findings; however, it appears that it did not impact our ability to saturate, at least within the chiropractic profession. Of those who consented to participate, 3/12 (25 %) were lost to follow-up. While we could not conclude reasons for loss to follow-up, this project was conducted during the COVID-19 pandemic, which may have played a role in participation [44]. Finally, potential bias may have occurred inadvertently through the research team’s assumptions and preconceptions. Reviewers did not specifically discuss how their personal beliefs and biases may have influenced the analyses of the data (reflexivity); however, we aimed to limit these biases through bracketing. First, the same experienced interviewer led all interviews using an interview guide, and a second researcher was present for all interviews. Second, three researchers independently coded themes. Finally, clinicians had the opportunity to review their transcripts and the overall thematic analyses.

## Conclusions

Through iKT, end-users can co-produce useful knowledge and knowledge tools. Exploring the nature of the end-user participation through their perceptions and reality may help support and strengthen future iKT methods; potentially improving knowledge uptake and patient health outcomes.

## Supplementary Information

**Additional file 1.** Interview Guide.

## Data Availability

The datasets during and/or analyzed during the current study available from the corresponding author on reasonable request.

## Abbreviations

iKT: Integrated knowledge translation
COREQ: Consolidated Criteria for Reporting Qualitative Research Checklist
CCGI: Canadian Chiropractic Guideline Initiative
