# Supplementary material for: Exploring clinicians’ experiences and perceptions of end-user roles in knowledge development: a qualitative study
Source: BMC Health Serv Res. 2021 Sep 6;21:926. doi: 10.1186/s12913-021-06955-7 (PMC8420847; doi:10.1186/s12913-021-06955-7)
Supplement: Supplementary file 1 — Additional file 1. Interview Guide. [file 12913_2021_6955_MOESM1_ESM.pdf]

Additional File 1: Interview Guide

Welcome

Welcome and thank you very much for agreeing to participate in this discussion. My name is ________ and my role is to ask you a few questions to get an understanding about your experience as a knowledge user engaged in this integrated knowledge translation (iKT) project. iKT is defined as an ongoing relationship between researchers and decision makers (e.g., clinicians, managers, policy-makers) for the purpose of engaging in a mutually beneficial study or research program to facilitate decision-making. We hope this process will help us understand how to better engage knowledge users to develop future guideline implementation tools.

Before we start, I will go over a few things mentioned in the consent form:

- Participation in this conversation is completely voluntary. If at any time you wish to leave the discussion, you may.
- You may choose to not answer any question.
- We will audio record the conversation to ensure that we capture all of your ideas.
- All of your answers are private. Your name, or any identifying information, will not appear on any of our analyses or reporting.

The discussion will last approximately 40 minutes.

Do you have any questions about what I just said?

Warm up question (for example): Can you tell me a bit about your practice and the patients you see?

1. Can you please describe your experience in being involved in creating the evidence-based care pathway for clinicians?
   a. PROBE: Did being a clinician help to inform the care pathway development? (compared to academic researchers only developing the care pathway). Why or why not?

2. What were your experiences with each of the iKT activities you participated in?
   a. PROBE: What were your thoughts of the online meetings with the other knowledge users?
   b. PROBE: What were your thoughts on the 'think aloud' activity? Do you think this was an appropriate activity to review the care pathway? Why or why not?
   c. PROBE: Do you feel like you have had an adequate opportunity to provide your feedback and share your ideas? Why or why not?

3. From your perspective, what role should a clinician have in developing clinical practice guidelines?
   a. PROBE: How important is it to you that clinicians in active practice participate in the development of these guidelines? Would this impact the credibility or applicability of the guidelines?

4. From your perspective, what role should a clinician have in developing guideline implementation tools?
   a. PROBE: How important is it to you that clinicians in active practice participate in the development of these tools? Would this impact the credibility or applicability of the tools?

5. What is your perspective on using iKT to develop the care pathway?
    a. PROBE: What were/are some of the enablers to this approach?
    b. PROBE: What were/are some of the barriers to this approach?

6. Would you suggest other iKT activities that you think may be useful in developing a care pathway to clinicians?
    a. PROBE: Why would you recommend these iKT activities? What are advantages/disadvantages of their use?

7. Do you have suggestions on how to improve our process of developing evidence-based care pathways in the future?
    a. PROBE: Would you recommend that we continue to use iKT to develop other guideline implementation tools such as other care pathways, patient handouts, or guideline summaries? Why or why not?

8. Given your involvement in developing the care pathway, will you use it in your practice?
    a. PROBE: What were/are some of the enablers to using it?
    b. PROBE: What were/are some of the barriers to using it?
    c. PROBE: Would you recommend the implementation of the care pathway in practice to colleagues? Why or why not?
    d. PROBE: Do you think your colleagues will use this care pathway? Why or why not?

Thank You/Wrap Up:
Thank you very much for sharing your ideas with us—we really appreciate your insights and time. Do you have anything else you'd like to tell me that we have not already discussed?

If you have any other questions following today's discussion, please feel free to contact me via email.
